# Acute kidney injury 2016: diagnosis and diagnostic workup

**DOI:** 10.1186/s13054-016-1478-z

**Published:** 2016-09-27

**Authors:** Marlies Ostermann, Michael Joannidis

**Affiliations:** 1 Department of Critical Care Medicine, King’s College London, Guy’s & St Thomas’ Foundation Hospital, Westminster Bridge Road, London, UK; 2 Division of Intensive Care and Emergency Medicine, Medical University of Innsbruck, Anichstr. 35, Innsbruck, Austria

## Abstract

Acute kidney injury (AKI) is common and is associated with serious short- and long-term complications. Early diagnosis and identification of the underlying aetiology are essential to guide management. In this review, we outline the current definition of AKI and the potential pitfalls, and summarise the existing and future tools to investigate AKI in critically ill patients.

## Background

Acute kidney injury (AKI) is a syndrome characterised by a rapid (hours to days) deterioration of kidney function. It is often diagnosed in the context of other acute illnesses and is particularly common in critically ill patients. The clinical consequences of AKI include the accumulation of waste products, electrolytes, and fluid, but also less obvious effects, including reduced immunity and dysfunction of non-renal organs (organ cross-talk) [1].

The impact and prognosis of AKI vary considerably depending on the severity, clinical setting, comorbid factors, and also geographical location. There is increasing evidence that AKI is associated with serious short- and long-term complications, in particular increased mortality and morbidity, the development of chronic kidney disease (CKD), and high financial healthcare costs. As such, AKI is now recognized as a major public health problem [2, 3].

Rapid diagnosis and appropriate diagnostic workup are essential to identify those types of AKI where specific therapies and interventions are available to reverse the injurious process within the kidneys. This review will summarise the key aspects of diagnosis and diagnostic work-up with particular focus on patients in the intensive care unit (ICU).

## Diagnosis of AKI

The diagnosis of AKI is traditionally based on a rise in serum creatinine and/or fall in urine output. The definition has evolved from the Risk, Injury, Failure, Loss, End-stage (RIFLE) criteria in 2004 to the AKI Network (AKIN) classification in 2007 [4, 5]. In 2012, both were merged resulting in the Kidney Disease Improving Global Outcomes (KDIGO) classification [6]. Accordingly, AKI is diagnosed if serum creatinine increases by 0.3 mg/dl (26.5 μmol/l) or more in 48 h or rises to at least 1.5-fold from baseline within 7 days (Table 1). AKI stages are defined by the maximum change of either serum creatinine or urine output. The importance of both criteria was confirmed in a recent study in >32,000 critically ill patients which showed that short- and long-term risk of death or renal replacement therapy (RRT) were greatest when patients met both criteria for AKI and when these abnormalities persisted for longer than 3 days [7].

**Table 1:** KDIGO definition and classification of AKI [6]

*Diagnostic criteria for AKI:*		
AKI is defined as any of the following:		
• Increase in serum creatinine by ≥0.3 mg/dl (≥26.5 μmol/l) within 48 h; or • Increase in serum creatinine to ≥1.5 times baseline, which is known or presumed to have occurred within the prior 7 days; or • Urine volume <0.5 ml/kg/h for 6 h.		
*AKI staging system:*		
AKI stage	Serum creatinine criteria	Urine output criteria
AKI stage I	Increase of serum creatinine by ≥0.3 mg/dl (≥26.4 μmol/L)	Urine output <0.5 ml/kg/h for 6–12 h
	or	
	increase to 1.5–1.9 times from baseline	
AKI stage II	Increase of serum creatinine to 2.0–2.9 times from baseline	Urine output <0.5 ml/kg/h for ≥12 h
AKI stage III	Increase of serum creatinine ≥3.0 times from baseline	Urine output <0.3 ml/kg/h for ≥24 h
	or	or
	serum creatinine ≥4.0 mg/dl (≥354 μmol/L)	anuria for ≥12 h
	or	
	treatment with RRT	
	or	
	in patients <18 years, decrease in estimated GFR to <35 ml/min per 1.73 m^2^	

*AKI* acute kidney injury, *GFR* glomerular filtration rate, *KDIGO* Kidney Disease Improving Global Outcomes, *RRT* renal replacement therapy

Several studies in various different patient populations have confirmed an association between stages of AKI and short- and long-term outcomes [8–13]. However, serum creatinine and urine output are markers of excretory function only and do not provide any information about any other roles of the kidney, i.e. metabolic, endocrine, or immunological functions. They are also not kidney specific and need to be interpreted within the clinical context. Some patients fulfil the AKI definition but do not have AKI, and there are also patients with clear evidence of renal injury who do not meet the creatinine or urine criteria for AKI [14, 15] (Table 2).

**Table 2:** Potential pitfalls of AKI diagnosis based on creatinine and urine criteria

Clinical scenario	Consequence
Administration of drugs which interfere with tubular secretion of creatinine (i.e. cimetidine, trimethoprim)	Misdiagnosis of AKI (rise in serum creatinine without change in renal function)
Reduced production of creatinine (i.e. muscle wasting, liver disease, sepsis)	Delayed or missed diagnosis of AKI
Ingestion of substances which lead to increased generation of creatinine independent of renal function (i.e. creatin, cooked meat)	Misdiagnosis of AKI
Obesity	Overdiagnosis of AKI if using actual weight when applying urine output criteria
Conditions associated with physiologically increased GFR (i.e. pregnancy)	Delayed diagnosis of AKI
Interference with analytical measurement of creatinine (i.e. 5-fluorocytosine, cefoxitin, bilirubin)	Misdiagnosis and delayed diagnosis of AKI (depending on the substance)
Fluid resuscitation and overload	Delayed diagnosis of AKI (dilution of serum creatinine concentration)
Progressive CKD with gradual rise in serum creatinine	Misdiagnosis of AKI
Extrinsic creatinine administration as a buffer in medications (i.e. in dexamethasone, azasetron)	Pseudo-AKI
Oliguria due to acute temporary release of ADH (i.e. post-operatively, nausea, pain) enhanced by maximal sodium reabsorption in the setting of volume/salt depletion	Misdiagnosis of AKI

*ADH* anti-diuretic hormone, *AKI* acute kidney injury, *CKD* chronic kidney disease, *GFR* glomerular filtration rate

### Limitations of creatinine-based criteria for AKI

**Fig. 1:**
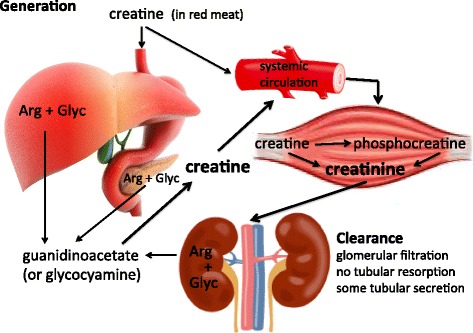
Generation and clearance of creatinine. *Arg* arginine, *Glyc* glycine

Serum creatinine is a metabolite of creatine, a molecule that is synthesized from the amino acids glycine and arginine in liver, pancreas, and kidneys and that serves as a rapidly mobilizable reserve of high-energy phosphates in skeletal muscle (Fig. 1). Creatinine production is determined by the amount of creatine generated in liver, pancreas, and kidneys, creatine ingested (i.e. intake of red meat) and muscle function. With a molecular weight of 113 Da, creatinine is freely filtered  by the glomeruli. In health, it is produced at a constant rate and the rate of production is matched by the rate of renal excretion. However, large and sustained falls in production have been demonstrated during critical illness [16–18].

The role of creatinine as a marker of renal function is limited by the fact that its half-life increases from 4 h to 24–72 h if the glomerular filtration rate (GFR) decreases. As such, the serum concentration may take 24–36 h to rise after a definite renal insult. Furthermore, a true fall in GFR may not be adequately reflected by serum creatinine in patients with sepsis, liver disease, and/or muscle wasting [15, 17, 18]. Serum creatinine concentrations are also affected by drugs which compete with tubular secretion. In this case, serum creatinine levels may fluctuate without a change in renal function (Table 2). There is also no standardized laboratory method for quantifying serum creatinine, and substances like bilirubin or drugs may interfere with certain analytical techniques, more commonly with Jaffe-based assays.

Serum creatinine is measured as a concentration and is therefore affected by variations in volume status. As a result, the diagnosis of AKI may be delayed or missed in patients with significant fluid shifts or fluid overload [19, 20]. This was highlighted in a post-hoc analysis of the Fluid and Catheter Treatment Trial [20]. It revealed that AKI was unmasked or classified differently in up to 18 % of patients after serum creatinine levels were adjusted for net fluid balance and estimated total body water. Affected patients had mortality rates similar to those with AKI that was present before adjustment.

Another important limitation of all creatinine-based definitions of AKI is that they require a reference value to describe “baseline” renal function. Ideally, this value should reflect the patient’s steady-state kidney function just before the episode of AKI. However, information on pre-hospital kidney function is not always available so that various surrogate estimates are frequently used. These may include inpatient results or the imputation of values such as back-calculating a baseline creatinine and using an estimated glomerular filtration rate (eGFR) of 75 ml/min per 1.73 m^2^ in patients with missing data [15]. Unfortunately, these methods can inflate as well as reduce the true incidence of AKI [21–23]. At present, there is no standard approach to determining baseline renal function.

Creatinine-based criteria for AKI often do not take into account underlying renal reserve. In patients with normal kidney function, a rise in serum creatinine by 0.3 mg/dl may indeed be due to an important reduction in GFR. In contrast, in patients with underlying CKD, absolute rises in serum creatinine represent variable changes in GFR, and a rise by 0.3 mg/dl may be within the acceptable daily variation and simply reflect an inconsequential change in GFR [24]. This is particularly relevant when diagnosing KDIGO AKI stage 3 which is defined by a rise in serum creatinine to >4.0 mg/dl (≥353.6 μmol/l). A patient with a baseline serum creatinine of 3.9 mg/dl (345 μmol/l) who experiences a creatinine rise by 0.3 mg/dl in 48 h would be classified as having KDIGO AKI stage 3, whereas such a rise would be defined as AKI stage 1 in a patient with normal baseline renal function [14].

Similar problems may occur when defining AKI stage 3 by the RRT criterion. The optimal timing of RRT for AKI is not known and clinical practice is very variable. As such, AKI staging depends directly on the decision-making process of the clinician rather than underlying renal function.

Finally, single serum creatinine values do not provide any information about specific stages of the AKI process. Importantly, they do not indicate whether a patient is still in the progression phase or if recovery has already begun. Also, eGFR formulae are not valid to determine renal function in AKI.

### Limitations of urine-based criteria for AKI

Urine output is an important clinical marker [7, 25] but, like creatinine, is not renal specific. In fact, urine output may persist until renal function almost ceases. Similarly, oliguria may be an appropriate physiological response of functioning kidneys during periods of prolonged fasting, hypovolaemia, after surgery, and following stress, pain, or trauma [26–28]. In these situations, the action of anti-diuretic hormone (ADH) can result in the generation of very concentrated urine with osmolarities up to 1400 mmosm/l. Assuming a daily solute load of 700 mosmoles, the urine volume may physiologically decrease to 500 ml (i.e. 0.28 ml/kg/h in a 70 kg person) as a result of normal kidney function [28].

The KDIGO criteria for AKI are based on the presence of oliguria for a minimum of 6 h [6]. Several experts have questioned the validity of this arbitrary cut-off and suggest using either a longer minimum period (e.g. 12 h) or a lower threshold for urinary output (e.g. 0.3 ml/kg/h instead of 0.5 ml/kg/h) to reach sufficient specificity for diagnosing AKI [14, 29].

Finally, in obese patients, weight-based urine output criteria may be particularly misleading (Table 2). In fact, the European Renal Best Practice Guidelines (2012) recommend using the ideal weight rather than the true weight when calculating urine output in ml/min/kg to avoid an overdiagnosis of AKI [30].

### Adjunctive diagnostic tools to diagnose AKI

In certain circumstances, it may be necessary to use additional tools to diagnose AKI, especially where creatinine and urine values change only slowly, are misleading, or cannot be interpreted accurately. This is particularly relevant for critically ill patients where the presence of fluid overload, muscle wasting, sepsis, and reduced effective circulating volume may completely mask the diagnosis of AKI.

#### New AKI biomarkers

**Fig. 2:**
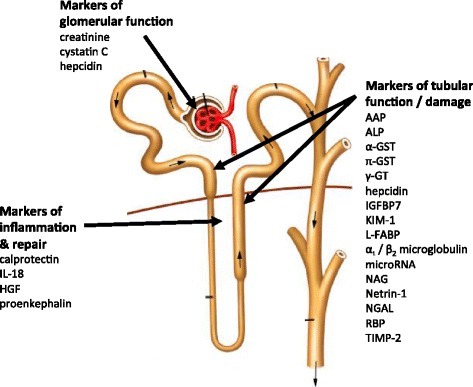
Biomarkers of AKI. *α-GST* α glutathione S-transferase, *AAP* alanine aminopeptidase, *ALP* alkaline phosphatase, *γ-GT* γ-glutamyl transpeptidase, *п GST* п glutathione S-transferase, *HGF* hepatocyte growth fator, *IGFBP-7* insulin like growth factor binding protein 7, *IL-18* interleukin 18, *KIM-1* kidney injury molecule-1, *L-FAB* liver fatty acid-binding protein, *NAG* N-acetyl-β-d-glucosaminidase, *NGAL* neutrophil gelatinase-associated lipocalin, *RBP* retinol binding protein, *TIMP2* tissue inhibitor metalloproteinase 2

Significant progress has been made in the detection and validation of new biomarkers for AKI to replace or complement serum creatinine. They vary in their anatomical origin, physiological function, time of release after the onset of renal injury, kinetics, and distribution [24, 25] (Table 3, Fig. 2). In addition to diagnosing AKI earlier, some of them may also provide information about the underlying aetiology and indicate different stages of the pathophysiological processes involved in AKI from acute injury to recovery [31].

**Table 3:** New diagnostic biomarkers of AKI evaluated in human studies

AKI biomarker	Description	Handling by the kidney	Factors affecting biomarker levels
Alanine aminopeptidase (AAP)	Enzymes located on the brush border villi of the proximal tubular cells	Released from tubular brush border after damage to proximal tubular cells	
Alkaline phosphatase (ALP)			
γ-Glutamyl transpeptidase (γ-GT)			
Angiopoietin-1	57 kDa endothelial growth factor secreted by endothelial cells, including renal endothelial cells	Upregulated in glomerular disease and sepsis	Systemic inflammation
Angiopoietin-2			
			Diabetes
			Malignancy
Calprotectin	Cytosolic calcium-binding complex of two proteins of the S100 group (S100A8/S100A9); derived from neutrophils and monocytes; activator of innate immune system	Detectable in urine following intrinsic AKI	Inflammatory bowel disease
			Urinary tract infection
			CKD
Chitinase 3-like protein 1	39 kDa soluble intracellular protein of glycoside hydrolase family 18 expressed by chrondrocytes, macrophages, endothelial cells, neutrophils, smooth muscle, and cancer cells;	Glomerular filtration of serum concentrations; in addition: some secretion by macrophages within the kidneys upon renal stress or damage	Inflammatory diseases
			Malignancy
			COPD
			Liver cirrhosis
			Connective tissue disease
			Cardiovascular disease
Cystatin C	13 kDa cysteine protease inhibitor produced by all nucleated human cells and released into the plasma at a constant rate	Freely filtered in glomeruli and completely absorbed and catabolized by proximal tubular cells; no tubular resorption or secretion	Systemic inflammation
			Malignancy
			Thyroid disorders
			Glucocorticoid disorder
			Cigarette smoking
			Hyperbilirubinaemia
			Hypertriglyceridaemia
			HIV disease
α Glutathione S-transferase (α GST)	47–51 kDa cytoplasmic enzyme in proximal tubule	Released into urine following tubular injury	
п Glutathione S-transferase (п GST)	47–51 kDa cytoplasmic enzyme in distal tubules	Released into urine following tubular injury	
Hepatocyte growth factor (HGF)	Antifibrotic cytokine produced by mesenchymal cells and involved in tubular cell regeneration after AKI	Released into urine following tubular injury	Advanced heart failure
			Hypertension
			Bowel inflammation
Hepcidin	2.78 kDa peptide hormone predominantly produced in hepatocytes but also in kidney, brain, and heart; regulator of iron metabolism	Freely filtered followed by tubular uptake and catabolism	Systemic inflammation
			Iron overload
Insulin-like growth factor binding protein-7 (IGFBP-7), tissue metalloproteinase-2 (TIMP-2)	Metalloproteinases involved in cell cycle arrest	Released into urine after tubular cell stress	
Interleukin-18 (IL-18)	18 kDa pro-inflammatory cytokine	Released into urine by proximal tubular cells following tubular injury	Inflammation
			Sepsis
			Heart failure
Kidney Injury Molecule-1 (KIM-1)	Transmembrane glycoprotein produced by proximal tubular cells after ischaemic or nephrotoxic injury	Released into urine following ischaemic or nephrotoxic tubular damage	Renal cell carcinoma
			Chronic proteinuria
			CKD
			Sickle cell nephropathy
Liver-type fatty acid-binding protein (L-FABP)	14 kDa intracellular lipid chaperone produced in proximal tubular cells and hepatocytes	Freely filtered in glomeruli and reabsorbed in proximal tubular cells; increased urinary excretion after tubular cell damage	CKD
			Polycystic kidney disease
			Liver disease
			Sepsis
α_1_ Microglobulin	Low molecular weight protein produced in liver	Freely filtered by glomeruli; reabsorbed and catabolised by proximal tubular cells; urinary excretion after tubular dysfunction	Sepsis
β_2_ Microglobulin	12 kDa light chain of major histocompatibility class I expressed on cell surface of every nucleated cell	Freely filtered by glomeruli; reabsorbed and catabolised by proximal tubular cells; urinary excretion after tubular dysfunction	
MicroRNA	Endogenous single-stranded molecules of non-coding nucleotides	Upregulated following tubular injury and detectable in plasma and urine	Sepsis
Monocyte chemoattractant peptide-1 (MCP-1)	Peptide expressed in renal mesangial cells and podocytes	Released into urine	Variety of primary renal diseases
N-acetyl-β-d-glucosaminidase (NAG)	>130 kDa lysosomal enzyme; produced in proximal and distal tubular cells and non-renal cells	Too large to undergo glomerular filtration; released into urine after tubular damage	Diabetic nephropathy
Neutrophil gelatinase-associated lipocalin (NGAL)	At least three different types:	25 kDa and 45 kDa NGAL undergo glomerular filtration and reabsorption in healthy tubular cells	Sepsis
	• Monomeric 25 kDa glycoprotein produced by neutrophils and epithelial tissues, including renal tubular cells • Homodimeric 45 kDa protein produced by neutrophils • Heterodimeric 135 kDa protein produced by renal tubular cells		Malignancy
			CKD
		25 kDa and 135 kDa NGAL are released into urine following tubular damage	Urinary tract infection
			Pancreatitis
			COPD
			Endometrial hyperplasia
Netrin-1	50–75 kDa laminin-related molecule, minimally expressed in proximal tubular epithelial cells of normal kidneys	Highly expressed in injured proximal tubules and released into urine	
Proenkephalin	Endogenous polypeptide hormone in adrenal medulla, nervous system, immune system and renal tissue	Cleared by glomerular filtration	Systemic inflammation
			Pain
Retinol binding protein (RBP)	21 kDa single-chain glycoprotein; synthesized by liver	Totally filtered by the glomeruli and reabsorbed but not secreted by proximal tubules; released into urine following tubular injury	Diabetes
			Obesity
			Acute critical illness
Soluble triggering receptor expressed on myeloid cells-1 (sTREM-1)	Member of the immunoglobulin superfamily of receptors expressed on granulocytes and monocytes, also possibly produced by endothelial cells and tubular epithelial cells	Detectable in urine following glomerular filtration and possibly local production	Sepsis
			Systemic inflammation

*AKI* acute kidney injury, *CKD* chronic kidney disease, *COPD* chronic obstructive pulmonary disease, *GFR* glomerular filtration rate, *HIV* human immunodeficiency virus

Biomarkers for AKI can be stratified into markers primarily reflecting glomerular filtration (i.e. serum cystatin C), glomerular integrity (i.e. albuminuria and proteinuria), tubular stress (i.e. insulin-like growth factor binding protein 7 (IGFBP-7), tissue inhibitor metalloproteinase 2 (TIMP2)), tubular damage (i.e. neutrophil gelatinase-associated lipocalin (NGAL), kidney injury molecule-1 (KIM-1), N-acetyl-β-d-glucosaminidase (NAG), liver fatty acid-binding protein (L-FAB)), and intra-renal inflammation (i.e. interleukin-18) [32–37] (Table 3, Fig. 2).

The availability of these new markers has allowed the detection of subtle changes in renal function before serum creatinine rises and the identification of patients with evidence of kidney injury without a change in serum creatinine, i.e. “sub-clinical AKI” [34, 35, 38–40]. Of note, biomarker-positive, creatinine-negative patients appear to have a greater risk of complications, a longer stay in hospital and higher mortality compared to patients without a biomarker rise [38]. However, in certain situations, these events reflect higher severity of illness rather than degree of AKI [41].

**Fig. 3:**
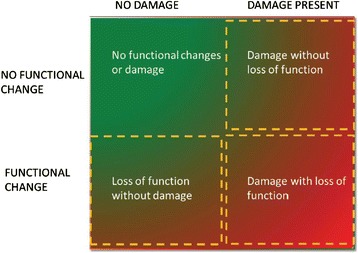
Diagnosis of AKI based on functional and damage markers. The combination of functional and damage biomarkers allows the clinician to diagnose AKI earlier and to differentiate the disease process better. It is recognised that the process is dynamic and that patients may move from one phase to another. Reproduced with permission from http://www.adqi.org/

The 10^th^ Acute Dialysis Quality Initiative (ADQI) Consensus Conference proposed to utilise both function and damage biomarkers in combination with traditional markers of renal function to better define and characterise AKI [35, 40] (Fig. 3). This approach appears to delineate the spectrum of AKI better than serum creatinine and urine output alone and has the potential to transform the way clinicians diagnose and manage patients with AKI.

Commercial kits for measurement of cystatin C, NGAL, IGFBP7 and TIMP-2 are available. To date, only cystatin C is routinely used in some hospitals. Cystatin C is a low molecular 13-kD inhibitor of lysosomal proteinases and extracellular inhibitor of cysteine proteases. It is produced in all nucleated cells and can be found in all tissues and body fluids. It is freely filtered in the glomeruli and then fully absorbed by the tubular cells and broken down. Since there is no tubular resorption or secretion, it is considered a better marker of GFR than serum creatinine. The main strength is that cystatin C is less dependent on age, gender, muscle mass, and liver function [34, 42]. However, cystatin C levels have been reported to be altered in some patients with cancer, thyroid dysfunction, or steroid therapy, and smokers [43–46].

## Diagnosis of acute kidney disease

AKI is defined as occurring over 7 days and CKD starts when kidney disease has persisted for more than 90 days. Based on epidemiological studies and histological case series, it is clear that some patients have a slow but persistent (creeping) rise in serum creatinine over days or weeks but do not strictly fulfil the consensus criteria for AKI [47, 48]. To classify this phase between the early stage of AKI (first 7 days) and the onset of CKD (beyond 3 months), the KDIGO expert group proposed the term “acute kidney disease” (AKD) and suggested the following criteria: a GFR <60 ml/min/1.73 m^2^ for <3 months, a decrease in GFR by ≥35 %, and an increase in serum creatinine by >50 % for <3 months or evidence of structural kidney damage for <3 months [6]. These criteria are currently under revision (personal communication with the ADQI group).

## Diagnostic work-up

As a syndrome, AKI can have multiple aetiologies. In critically ill patients, the most common causes are sepsis, heart failure, haemodynamic instability, hypovolaemia, and exposure to nephrotoxic substances [9]. Acute parenchymal and glomerular renal diseases are relatively rare. Determining the aetiology is essential to guide management and potentially target and influence the disease process.

The terms “pre-renal”, “renal” and “post-renal” have traditionally been used to narrow the differential diagnosis of AKI. It was a long-held view that “pre-renal AKI” or “transient” AKI were synonymous with “hypovolaemic AKI” and “fluid responsiveness” [49]. However, several studies have demonstrated that tubular damage may be present in patients with “pre-renal AKI” [50, 51]. Furthermore, adverse outcomes have been noted even when creatinine returned to baseline within 24 h [52]. Based on these results, the ADQI group proposed to differentiate between “functional AKI” and “kidney damage” in preference to the terms “pre-renal”, “renal”, and “post-renal” AKI [49].

**Fig. 4:**
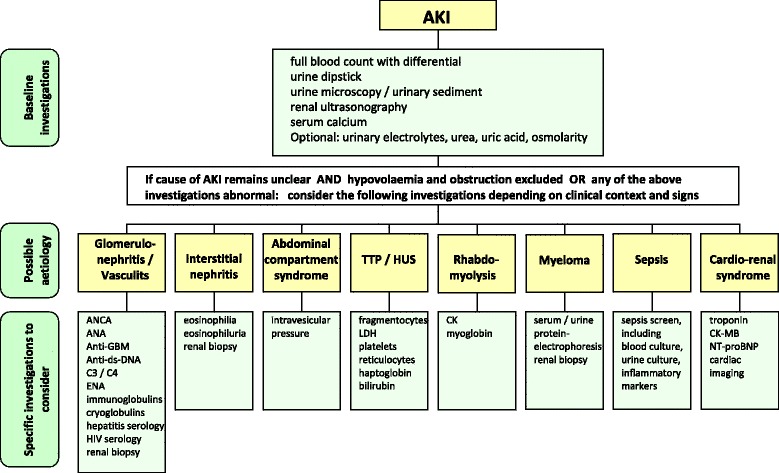
Diagnostic work up. *AKI* acute kidney injury, *ANCA* anti-neutrophil cytoplasmic antibody, *ANA* anti-nuclear antibody, *Anti-ds-DNA* anti-double stranded DNA, *anti-GBM* anti-glomerular basement membrane, *C3* complement component 3, *C4* complement component 4, *CK* creatine kinase, *CK-MB* creatine kinase MB fraction, *ENA* extractable nuclear antigen, *HIV* human immunodeficiency virus, *HUS* haemolytic uraemic syndrome, *LDH* lactate dehydrogenase, *NT-proBNP* N-terminal pro-brain natriuretic peptide, *TTP* thrombotic thrombocytopenic purpura

The specific diagnostic workup in individual patients with AKI depends on the clinical context, severity, and duration of AKI, and also on local availability. Urinalysis, examination of the urinary sediment, and imaging studies should be performed as a minimum, with additional tests depending on the clinical presentation (Fig. 4).

### Urine dipstick

Urine dipstick testing is a simple test to undertake. In fact, the AKI guideline by the National Institute for Health and Care Excellence (NICE) in the UK recommends performing urine dipstick testing for blood, protein, leucocytes, nitrites, and glucose in all patients as soon as AKI is suspected or detected in order not to miss any potentially treatable glomerular or tubular pathologies [53]. These include:

- glomerulonephritis (with haematuria and proteinuria)
- acute pyelonephritis (with pyuria/leucocyturia and nitrites in urine)
- interstitial nephritis (occasionally with eosinophiluria)

It is important to consider the result of the urine dipstick alongside the clinical history and an evaluation of the patient. For instance, the presence of white blood cells is non-specific but may indicate an underlying infection or acute interstitial nephritis. Similarly, dipstick haematuria in a patient with an indwelling urinary catheter can have multiple aetiologies ranging from glomerulonephritis to simple trauma. Dipsticks detect haemoglobin and remain positive even after red cell lysis. They also detect haemoglobinuria from intravascular haemolysis as well as myoglobin from muscle breakdown. A urine dipstick positive for haemoglobin without red blood cell positivity suggests a possible diagnosis of rhabdomyolysis.

### Urine microscopy (urinary sediment)

Urine microscopy can provide very valuable information when performed by a skilled operator using a freshly collected non-catheterised urine sample (Table 4). It is not utilized very often in the ICU predominantly because it is operator dependent and requires training and experience. When done properly, the presence of red cell casts or dysmorphic red cells supports the diagnosis of glomerular disease [54–58]. Urine microscopy may also help to diagnose septic AKI and predict worsening renal function. Bagshaw and colleagues collected blood and urine samples of 83 critically ill patients with sepsis of whom 52 % had AKI [55]. They derived a urine microscopy score based on the observed quantification of renal tubular epithelial cells and granular casts in the sediment and showed that septic AKI was associated with greater urine microscopy evidence of kidney injury compared with non-septic AKI, despite similar severity of AKI. A higher urine microscopy score was also predictive of worsening AKI. Finally, urine microscopy can be informative in rare cases of AKI; for instance, ethylene glycol poisoning where oxalate crystals may be seen, in case of tumour lysis syndrome where urate crystals may be present, or in light chain disease.

**Table 4:** Interpretation of urine microscopy findings

Microscopy finding	Example	Significance
Epithelial cells		Normal
Renal tubular cells		Acute tubular injury
Non-dysmorphic red cells		Non-glomerular bleeding from anywhere in the urinary tract
Dysmorphic red cells		Glomerular disease, but can also be seen if urine sample is not fresh at time of microscopy
Red cell casts		Diagnostic of glomerular disease
Leukocytes		Up to 3 per high-power field = normal; >3 per high-power field = inflammation in urinary tract
White cell casts		Renal infection
Hyaline casts		Any type of renal disease
Granular casts		More significant renal disease
“Muddy brown cast”		Necrotic tubular cells aggregated with tamm horsfall protein indicating acute tubular injury
Crystals		Some crystals can be found in healthy individuals; “abnormal” crystals may indicate metabolic disorders or excreted medications
Bacteria		Urinary tract infection; contamination

### Urinary electrolytes

Measurement of urinary electrolytes and fractional excretion of sodium (FENa), urea, or uric acid has not been consistently shown to have clear correlations with clinical and histopathological findings [54, 59, 60]. In situations associated with transient hypovolaemia or hypoperfusion, healthy kidneys respond by increasing urine osmolarity and reducing sodium and/or urea or uric acid excretion. However, this physiological response may be variable and confounded by CKD and co-interventions, including diuretic therapy, aminoglycosides, and cardiopulmonary bypass [60–64]. Whereas the presence of low fractional sodium (<1 %), uric acid (<12 %), and urea excretion (<34 %) together with a normal urinary sediment may support the diagnosis of functional AKI, the absence of these typical urinary electrolyte abnormalities would not exclude it [65, 66]. Finally, low FENa values have also been observed in experimental sepsis with increased renal blood flow as well as in the first hours of sepsis in humans [67–69].

As such, the interpretation of urinary electrolytes is challenging [70]. A single measurement of urinary electrolytes has a limited role in determining the differential diagnosis of AKI in critically ill patients. Instead, serial monitoring of urinary electrolytes may be more useful as sequential alterations in urine composition have been shown to parallel the development and severity of AKI [71, 72]. However, whether serial measurement of urine electrolytes can also help diagnosing the aetiology of AKI remains unclear.

### Renal ultrasound

Renal ultrasonography is useful for evaluating existing structural renal disease and diagnosing obstruction of the urinary collecting system. In particular, the presence of reduced corticomedullary differentiation and decreased kidney size is indicative of underlying CKD. In patients with abdominal distension ultrasonography can be technically challenging, in which case other imaging studies will be necessary.

Renal Doppler ultrasound and contrast-enhanced ultrasound are two relatively new techniques that may be used at the bedside to estimate renal perfusion and renal cortical microcirculation, respectively [73–75]. The non-invasiveness, repeatability, and accessibility of these techniques appear promising, but broad clinical use is still limited by training requirements as well as uncertainty how to interpret the information obtained. Finally, although Doppler scans may detect the presence of reduced renal blood flow, they are of little use to determine the specific aetiology of AKI.

### Measurement of intra-abdominal pressure

In case of suspected AKI due to intra-abdominal compartment syndrome, serial measurement of intra-abdominal pressure should be considered. Those with a pressure rise to >20 mmHg should be suspected of having AKI as a result of intra-abdominal compartment syndrome [76].

### Autoimmune profile

Depending on the clinical context, clinical signs, and urine dipstick results, patients may require specific immunological tests, including anti-neutrophil cytoplasmic antibody (ANCA), anti-nuclear antibody (ANA), anti-glomerular basement membrane antibody (anti-GBM), and complement component 3 and 4 to rule out immune-mediated diseases (i.e. vasculitis, connective tissue diseases) (Fig. 4). These investigations should be considered mandatory in patients with AKI presenting primarily with a pulmonary-renal syndrome, haemoptysis, or haemolysis/thrombocytopenia.

### Renal biopsy

Renal biopsies are rarely performed in critically ill patients, mainly due to the perceived risk of bleeding complications and general lack of therapeutic consequences. However, a renal biopsy may offer information that is not available through other means and should be considered if underlying parenchymal or glomerular renal disease is suspected (Fig. 4). Interestingly, Chu et al. reported that diffuse histological changes of AKI could be present without a sufficient change in serum creatinine [47]. Among 303 patients with biopsy-proven acute parenchymal renal lesions, including crescentic glomerulonephritis and acute thrombotic microangiopathy, only 198 patients (65 %) met the KDIGO creatinine or urine criteria for AKI. In a separate study from France, about 50 % of patients with AKI undergoing renal biopsy had a diagnosis distinct from acute tubular necrosis that frequently resulted in a change of treatment regimen [77]. Recent reports have suggested that transjugular renal biopsies may be safer than percutaneous or open techniques [78].

### Other laboratory tests

Depending on the clinical context, the following tests may be indicated:

- serum creatine kinase and myoglobin (in case of suspected rhabdomyolysis)
- lactate dehydrogenase (LDH) (in case of suspected thrombotic thrombocytopenic purpura (TTP))
- fragmentocytes (in case of possible TTP/haemolytic uraemic syndrome (HUS))
- N-terminal pro-brain natriuretic peptide (NT-proBNP) and troponin (in case of suspected cardio-renal syndrome)
- serum/urine protein electrophoresis (in case of suspected myeloma kidney)

### Challenges of diagnosing AKI in critically ill patients

As outlined earlier, the use of serum creatinine to estimate GFR in critically ill patients is limited by the lack of steady-state conditions, unpredictable rate of production, and variable degree of elimination (Table 2). Medications may cause increases in creatinine without reflecting a true decrease in GFR and fluid overload may lead to a dilution of creatinine concentrations. Finally, serum creatinine substantially lags behind a reduction in GFR and thus does not provide a useful real-time assessment of GFR. It is therefore not surprising that AKI is often diagnosed late in critically ill patients.

The interpretation of additional diagnostic investigations can be challenging, too. Dipstick haematuria is not uncommon in patients with an indwelling urinary catheter and most commonly due to simple trauma. Even more specialised tests, like autoimmune tests, have a higher risk of false-positive results in critically ill patients. For instance, infection is a frequent cause of a false-positive ANCA result [79]. Until more reliable tests are routinely used in clinical practice it is essential to interpret creatinine results and other diagnostic tests within the clinical context [80].

### Future diagnostic tools

A variety of new functional and damage markers of AKI have been shown to provide information related to the underlying pathophysiology of AKI and may also be utilised as diagnostic tools. It is expected that some of these markers will be routinely integrated into the definition as well as diagnostic workup of AKI [49].

Achieving the ability to rapidly and accurately measure and monitor GFR in real time would be very beneficial, especially in the ICU [81, 82]. Several groups are developing optical measurement techniques using minimally invasive or non-invasive techniques that can quantify renal function independent of serum creatinine or urine output. In the past few years, significant progress has been made in using two-photon excitation fluorescence microscopy to study kidney function [82]. It is very likely that several of these approaches will enter clinical phase studies in the very near future. These techniques will enable an earlier diagnosis of AKI and also provide opportunities to improve clinical management, including the use of nephrotoxic substances and appropriate drug dosing.

New imaging techniques may also be utilised, including cine phase-contrast magnetic resonance imaging or intravital multiphoton studies [83, 84]. However, given the complexity, financial costs, and need for patient transport, it is likely that they will remain research tools.

## Conclusion

Acute kidney injury is a clinical syndrome defined by a rise in serum creatinine and/or fall in urine output as per KDIGO classification. Future definitions are likely to incorporate novel functional and damage biomarkers to characterise AKI better. Early diagnosis and appropriate diagnostic work-up are essential to determine the underlying aetiology and to identify cases of AKI that require specific and timely therapeutic interventions. The exact diagnostic investigations depend on the clinical context and should include routine baseline tests as well as more specific and novel tools.

## Abbreviations

ADH: Anti-diuretic hormone
ADQI: Acute Dialysis Quality Initiative
AKD: Acute kidney disease
AKI: Acute kidney injury
AKIN: Acute Kidney Injury Network
ANCA: Anti-neutrophil cytoplasmic antibody
ANA: Anti-nuclear antibody
Anti-GBM: Anti-glomerular basement membrane
CKD: Chronic kidney disease
eGFR: Estimated glomerular filtration rate
FENa: fractional excretion of sodium
GFR: Glomerular filtration rate
HUS: Haemolytic uraemic syndrome
ICU: Intensive care unit
IGFBP-7: Insulin-like growth factor binding protein 7
KDIGO: Kidney Disease Improving Global Outcomes
LDH: Lactate dehydrogenase
KIM-1: Kidney injury molecule-1
L-FAB: Liver fatty acid-binding protein
NAG: N-acetyl-β-D-glucosaminidase
NGAL: Neutrophil gelatinase-associated lipocalin
NICE: National Institute for Health Care and Excellence
NT-proBNP: N-terminal pro-brain natriuretic peptide
RIFLE: Risk, Injury, Failure, Loss, End-stage
RRT: Renal replacement therapy
TIMP2: Tissue inhibitor metalloproteinase 2
TTP: Thrombotic thrombocytopenic purpura
